# Biomimetically enhanced demineralized bone matrix for bone regenerative applications

**DOI:** 10.3389/fphys.2015.00292

**Published:** 2015-10-23

**Authors:** Sriram Ravindran, Chun-Chieh Huang, Praveen Gajendrareddy, Raghuvaran Narayanan

**Affiliations:** ^1^Departments of Oral Biology, University of Illinois at ChicagoChicago, IL, USA; ^2^Departments of Periodontics, University of Illinois at ChicagoChicago, IL, USA

**Keywords:** demineralized bone matrix, biomimetic materials, mesenchymal stem cells, bone regeneration and repair, extracellular matrix

## Abstract

Demineralized bone matrix (DBM) is one of the most widely used bone graft materials in dentistry. However, the ability of DBM to reliably and predictably induce bone regeneration has always been a cause for concern. The quality of DBM varies greatly depending on several donor dependent factors and also manufacturing techniques. In order to standardize the quality and to enable reliable and predictable bone regeneration, we have generated a biomimetically-enhanced version of DBM (BE-DBM) using clinical grade commercial DBM as a control. We have generated the BE-DBM by incorporating a cell-derived pro-osteogenic extracellular matrix (ECM) within clinical grade DBM. In the present study, we have characterized the BE-DBM and evaluated its ability to induce osteogenic differentiation of human marrow derived stromal cells (HMSCs) with respect to clinical grade commercial DBM. Our results indicate that the BE-DBM contains significantly more pro-osteogenic factors than DBM and enhances HMSC differentiation and mineralized matrix formation *in vitro* and *in vivo*. Based on our results, we envision that the BE-DBM has the potential to replace DBM as the bone graft material of choice.

## Introduction

Bone is the second most transplanted organ in the human body (Marino and Ziran, 2010). Bone grafting is used in several aspects of medicine ranging from placement of dental implants and spinal fusions to regenerating lost bone resulting from trauma or congenital anomalies. With respect to children, over 75% of birth defects are craniofacial anomalies (such as cleft palate) that require bone reconstruction procedures (Zuk, 2008). Finally, with the many wars around the globe, the incidence of injuries requiring bone reconstruction is at an all time high.

Over the past decade several natural and synthetic biomaterials have been developed to aid bone regeneration (George and Ravindran, 2010). However, they have not been able to replace currently used bone graft materials successfully. On the other hand, the focus of current research has shifted away from improving the performance of existing clinical materials. The most immediate clinical need lies in the generation of products that improve the performance of existing materials or modified versions of existing clinical materials with improved performance. The focus of this study is the generation of a modified bone graft material with superior osteoinductive properties.

The gold standard for bone regenerative procedures is autografts. In situations that require significant quantity of bone to be regenerated, donor site morbidity becomes an issue with autografts. Under these circumstances, bone graft materials such as allograft bone are used. DBM is a commonly used allograft bone material. It is most commonly used in appendicular, axial and craniofacial bone regenerative applications (Gruskin et al., 2012). However, The quality and effectiveness of commercial DBM varies with processing techniques and several donor dependent factors (Zhang et al., 1997; Schwartz et al., 1998; Lohmann et al., 2001; Traianedes et al., 2004). As a result, bone regeneration and augmentation procedures do not have predictable outcomes. Additionally, the osteoinductive and osteogenic capacity of allograft DBM is significantly lesser than autografts (Marino and Ziran, 2010).

Very few medical products have as much variability in composition and performance as commercially available clinical grade DBM. Several inconsistencies have been reported with respect to DBM: The presence of osteoinductive growth factors (such as BMPs) varies significantly between batches of DBM and even amongst samples from the same batch (Bae et al., 2006, 2010). DBM from female donors has been shown to contain higher quantities of BMPs (Pietrzak et al., 2006). DBM shows variability in performance depending on donor age (Schwartz et al., 1998) and finally revascularization of DBM and allograft bone is poor (Delloye et al., 2007). Recently, several approaches have been studied to enhance the effectiveness of DBM such as remineralization (Soicher et al., 2013; Horváthy et al., 2015)and use of platelet rich plasma (Han et al., 2009) with limited and varied results. DBM in combination with bone marrow aspirates has been used to treat bone cysts effectively (Park et al., 2008; Di Bella et al., 2010), but it is not as effective when used for fracture healing and large quantity bone regenerative applications (Drosos et al., 2015).

Clinically, in order to augment bone regeneration, recombinant BMP2 is used clinically. Although it is very potent, dosage issues and ectopic effects are major problems facing BMP2 usage. Many complications have been reported recently causing serious safety concerns among clinicians (Thibault et al., 2010; Zara et al., 2011).

We have attempted to solve the inherent problems associated with DBM by generating a biomimetically-modified version of clinical grade DBM. We have achieved this by incorporating the native extracellular matrix (ECM) of differentiating mesenchymal stem cells (MSCs) within it. We chose MSCs as the source cell for creating the ECM, as the resulting matrix would be a combination of MSC matrix and that of MSCs differentiating into osteoblasts. This can provide an ideal environment for patient specific MSCs. The other alternative would be osteoblasts. However, osteoblasts have limited proliferative ability and hence would not be able to populate the DBM as effectively as MSCs. We have published previously on the standardization of conditions and advantages of using biomimetic scaffolds for tissue regeneration (Ravindran et al., 2012, 2014b). We have used these techniques to develop a modified clinical product with enhanced performance. The aim of this study was to evaluate the ability of our biomimetically enhanced DBM (BE-DBM) in comparison with commercial DBM using *in vitro* and *in vivo* experimental models to analyze if the BE-DBM depicts improved stem cell attachment, proliferation and differentiation abilities.

## Materials and methods

### Cell culture

Human marrow stromal cells were used in this study (HMSCs). These primary cells were obtained from NIH-funded center for research resources, Tulane Center for the preparation and distribution of adult stem cells. The cells are representative of marrow stromal cells from five donors between the age groups of 19 and 29 consisting of both male and female donors (Sekiya et al., 2002). We have published previously using these cells and they have been verified for multipotetncy (Ravindran et al., 2012, 2014a, 2015). The cells were cultured in αMEM basal media (Gibco) with 20% FBS (Gibco), 1% L-glutamine (Gibco) and 1% antibiotic-antimycotic solution (Gibco).

### Generation of BE-DBM

BE-DBM was generated using clinical grade commercial DBM. Two different types of DBM were used to generate BE-DBM. For the *in vitro* experiments, we used DBM granules alone. For the *in vivo* experiments, we used DBM granules containing cortical bone chips to serve as internal control for mineralized matrix. For each of the two types of DBM, we used three vials to generate the BE-DBM. Once the BE-DBM was prepared, they were stored as lyophilized samples until required for experimental use.

BMSCs were seeded on to the DBM at a concentration of 1 million cells per 250 mg of DBM granules and cultured in standard BMSC culture media for a period of 24 h. After this, the cells were cultured for 2 weeks in osteogenic culture media (growth media containing100 μg/ml ascorbic acid, 10 mM β-glycerophosphate and 10 mM dexamethasone). This was performed to induce osteogenic differentiation of the BMSCs and to facilitate the generation of a pro-osteogenic matrix. After 2 weeks, the DBM granules containing BMSCs were decellularized using our published protocol (Ravindran et al., 2012, 2014a,b). Briefly, the samples were incubated for 1 h at 37°C in TBS containing 0.5% triton x-100. After this, the cells were lysed using 25 mM ammonium hydroxide solution. They were then washed extensively in TBS followed by one wash in HBSS. Finally, DNAse digestion was performed and the samples were washed in double deionized water extensively, lyophilized and stored. Decellularization was verified by immunostaining for tubulin and DAPI nuclear staining.

### Proliferation experiment

Twenty thousand HMSCs were seeded on to equal amounts of DBM and BE-DBM in quadruplicates. Eight hours post seeding, the unattached cells were removed by aspiration and the samples were washed using three exchanges of fresh media to ensure complete removal of all unattached cells. Twenty-four hours post seeding, the number of live cells was quantitatively measured using an MTT cell titer assay (Promega). The number of cells present at various time points up to 1 week was also measured to obtain the proliferation rate.

### Electron microscopy

Electron microscopic evaluation was performed on DBM and BE-DBM in their natural state as well as when subjected to *in vitro* mineralization. To generate mineralized DBM and BE-DBM, *in vitro* mineralization experiment was performed as per previously published protocols (Ravindran et al., 2014b). Electron microscopic evaluation of the samples was performed after coating the samples with platinum and palladium using Hitachi S3000 electron microscope. Energy dispersive X-ray (EDX) analysis in variable pressure mode was performed to obtain elemental information on the mineralized samples and also to obtain ratios of calcium to phosphorus in the samples. For all EDX analyses, the samples were not coated.

### *In vitro* differentiation of HMSCs

500,000 HMSCs were seeded on to 50 mg of DBM or BE-DBM and cultured in standard growth media for 2 weeks in quadruplicates. After 2 weeks, the RNA was isolated from both sets of samples. After first strand synthesis (BioRad first strand synthesis kit), quantitative real time PCR (qPCR) was performed using gene specific primers. Table 1 lists the primer sequences used in this study. All expression data were normalized to housekeeping genes GAPDH and B2M.

**Table 1:** **qPCR primer sequences**.

**Gene**	**Forward**	**Reverse**
FGF2	5′-AGA AGA GCG ACC CTC ACA TCA-3′	5′-CGG TTA GCA CAC ACT CCT TTG-3′
GDF10	5′-AGA TCG TTC GTC CAT CCA ACC-3′	5′-GGG AGT TCA TCT TAT CGG GAA CA-3′
PHEX	5′-GAG GCA CTC GAA TTG CCC T-3′	5′-ACT CCT GTT TAG CTT GGA GAC TT-3′
OCN	5′-AGC CCA TTA GTG CTT GTA AAG G-3′	5′-CCC TCC TGC TTG GAC ACA AAG-3′
VEGFA	5′-AGG GCA GAA TCA TCA CGA AGT-3′	5′-AGG GTC TCG ATT GGA TGG CA-3′
GAPDH	5′-CAG GGC TGC TTT TAA CTC TGG-3′	5′-TGG GTG GAA TCA TAT TGG AAC A-3′
B2M	5′-GAG GCT ATC CAG CGT ACT CCA-3′	5′-CGG CAG GCA TAC TCA TCT TTT-3′
DMP1	5′-CTC CGA GTT GGA CGA TGA GG-3′	5′-TCA TGC CTG CAC TGT TCA TTC-3′

*Table showing the forward and reverse primer sequences for the genes used in the real time PCR analyses*.

### *In vivo* implantation

All experiments were performed in accordance with approved animal care protocols (Assurance No: A3460-01). 500,000 HMSCs were seeded on to 50 mg of DBM or BE-DBM encapsulated in collagen sponges (Zimmer collagen tape) and implanted subcutaneously on the back of 2-month-old athymic male nude mice for a period of 2 weeks. Experiments were performed in triplicate. We had two groups one with commercial DBM and the other with BE-DBM. Two implants were placed on the back of each mouse. On one side, we placed the DBM implant and the other side contained the BE-DBM implant. Therefore, a total of three mice were used for this study. After 2 weeks, the samples were extracted, fixed in 4% neutral buffered formalin, paraffin embedded and sectioned.

### Histology and immunohistochemistry

Hematoxylin and eosin (H&E) staining and alizarin red staining was performed as per previously published protocols (Ravindran et al., 2014a). Expression of proteins in the explant sections as well as DBM and BE-DBM sections were performed either using peroxidase conjugated secondary antibodies (Vector Laboratories DAB kit) or using secondary antibodies conjugated to fluorescent probes. The following primary antibodies were used: rabbit polyclonal anti fibronectin antibody (1/250, abcam), mouse monoclonal anti tubulin antibody (1/1000, Sigma), mouse monoclonal anti bone morphogenetic protein 2 (BMP2) antibody (1/100, abcam), rabbit polyclonal anti transforming growth factor β1 (TGFβ) antibody (1/100, abcam), mouse monoclonal anti phosphorylated serine (pSer) antibody (1/100, Santa Cruz biotechnology), mouse monoclonal anti DMP-1 antibody (1/1000, a gift from Dr. Chunlin Qin, Baylor College of Dentistry), mouse monoclonal anti osteocalcin antibody (1/100, abcam), mouse monoclonal anti runt-related transcription factor x2 (RUNX2) antibody (1/100, abcam), mouse monoclonal anti von Willebrand factor antibody (1/100, Santa Cruz biotechnology), rabbit polyclonal anti VEGF antibody (1/100, abcam).

Fluorescent microscopy was performed using a Zeiss LSM 710 laser scanning confocal microscope or a BioRad Zoe fluorescent microscope. For all comparative samples, the imaging parameters were maintained constant. For peroxidase stained sections, microscopy was performed using a Zeiss Axio Observer D1 microscope or an EVOS XL Core microscope (Life Technologies).

### Quantitation of proteins in DBM and BE-DBM

ELISA was used to quantitate the amount of a few important pro-osteogenic proteins in the DBM and BE-DBM. Equal amounts of both materials (10 mg) were placed in 96 well plates in quadruplicates. The material was blocked with 5% BSA for 1 h and incubated with the appropriate primary antibody overnight at 4°C. The samples were then washed four times in PBS and incubated with a biotinylated secondary antibody (1/250, Vector Laboratories) and then subsequently washed four times with PBS and incubated with peroxidase conjugated avidin (Vector Laboratories) for 1 h at room temperature. Finally, the samples were incubated with an ELISA substrate (Turbo TMB ELISA substrate, Thermo Scientific) for 5 min at room temperature. The reaction was stopped by the addition of 1 M sulfuric acid. The solution was then transferred to empty 96 well plate wells and absorbance at 490 nm was recorded. The following primary antibodies were used: rabbit polyclonal anti fibronectin antibody (1/500), mouse monoclonal anti BMP2 antibody (1/250), rabbit polyclonal anti VEGF antibody (1/250), mouse monoclonal anti phosphorylated serine, threonine, and tyrosine antibody (1/1000, abcam).

### Statistical analysis

Student's *t*-test was performed for the indicated experiments to verify statistical significance. A *P* < 0.05 (95% confidence interval) was considered as statistically significant.

## Results

### Characterization of BE-DBM for enhanced presence of pro-osteogenic proteins

**Figure 1:**
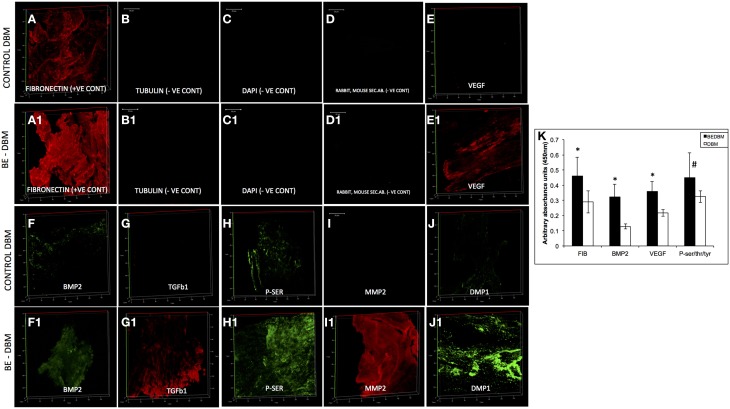
**ECM proteins in DBM and BE-DBM**. Images compare the expression of representative ECM proteins in DBM **(A–J)** and BE-DBM **(A1–J1)**. All images except **B,B1,C,C1,D**,**D1** (scale bar represents 20 μm for these images) are 3D renderings of z-stack confocal images. Tubulin **(B,B1)** was used as a negative control for absence of intracellular proteins and DAPI **(C,C1)** nuclear staining was used as negative control for absence of cellular DNA. Immunostaining was performed for Fibronectin **(A,A1)**, VEGF **(E,E1)**, BMP2 **(F,F1)**, TGFβ1 **(G,G1)**, proteins containing phosphorylated serines **(H,H1)** matrix metalloproteinase 2 (MMP2, **I,I1**) and DMP1 (**J,J1**). Panel **(K)** is a graphical representation of the relative amounts of select ECM proteins in both DBM and BE-DBM (*n* = 4). The quantified proteins were: Fibronectin (FIB), BMP2, VEGF, and phosphorylated proteins (P-ser/thr/tyr). Statistical significance was measured using student's *t*-test. ^*^Represents statistical significance with *P* < 0.05 and ^#^represents statistical significance with *P* < 0.1. Phosphorylated proteins were quantified using an antibody that recognizes phosphorylated serine, threonine and tyrosine residues.

DBM and BE-DBM sections were immunostained for the presence of several ECM proteins and growth factors that play an important role in osteogenesis and osteogenic differentiation of MSCs. Figure 1 shows representative images of 3D confocal microscopy. Results presented in Figure 1 show that the BE-DBM contains more pro-osteogenic proteins when compared to the commercially available DBM. The BE-DBM was generated using the DBM from the same container and hence, the results are directly comparable. In the results presented in Figure 1, tubulin was used as a negative control to show the absence of intracellular proteins (Figures 1B,B1). DAPI staining was performed to show the absence of cellular DNA (Figures 1C,C1). Rabbit and mouse secondary antibody controls were performed to show absence of non-specific staining (Figures 1D,D1).

The results presented in Figure 1 show that the BE-DBM contains more structural proteins such as fibronectin (Figure 1A vs. Figure 1A1), growth factors (Figures 1F,G vs. Figures 1F2,G1), pro-angiogenic factors such as VEGF (Figure 1E vs. Figure 1E1), phosphorylated proteins (Figure 1H vs. Figure 1H1) proteases (Figure 1I vs. Figure 1I1), and nucleating proteins (Figure 1J vs. Figure 1J1).

ELISA was used to quantify the increased presence of fibronectin, BMP2, VEGF, and phosphorylated proteins. The graph presented in Figure 1K shows a statistically significant increase in the presence of these proteins within the BE-DBM when compared to DBM.

### Electron microscopic evaluation of DBM and BE-DBM

**Figure 2:**
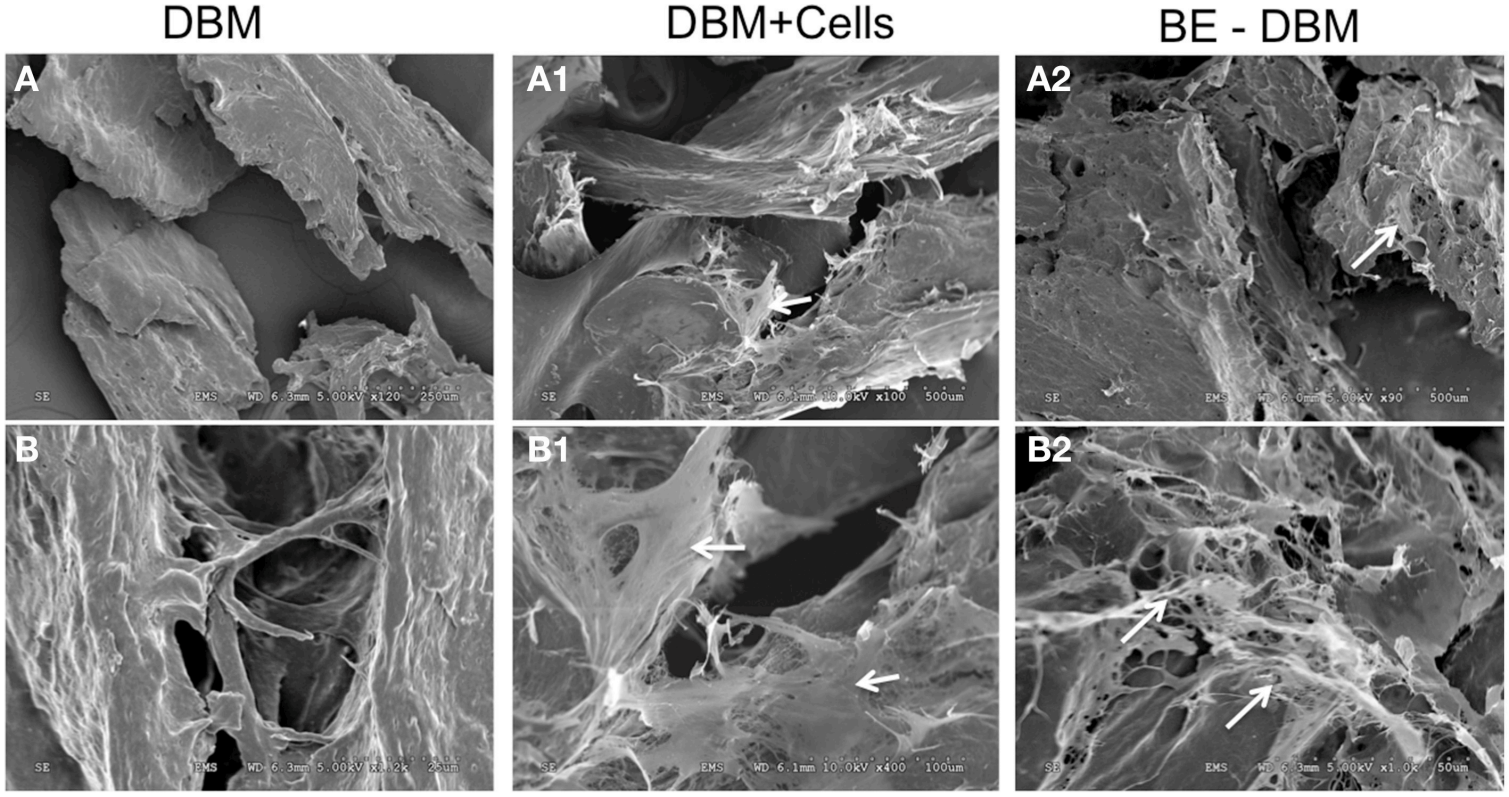
**Electron microscopic evaluation of DBM and BE-DBM**. Images are representative electron micrographs of DBM **(A,B)**, DBM with HMSCs **(A1,B1)** and BE-DBM **(A2,B2)**. Panels **(A,A1,A2)** are low magnification images. Panels **(B–B2)** are higher magnification images showing the microstructure. The white arrows in **(A1,B1)** point to the cells. White arrows in **(A2,B2)** point to ECM deposition in the BE-DBM samples. Note the absence of such structures in images **(A,B)**.

The microstructure of DBM and BE-DBM was evaluated using electron microscopy. Figure 2 represents results from this experiment. Figures 2A,B are images of commercial DBM. Figures 2A1,B1 show the presence of HMSCs on the DBM before decellularization is performed. Figures 2A2,B2 show images of BE-DBM containing the cell-secreted ECM. Upon comparing Figures 2A,B pertaining to DBM with Figure 2A2 and Figure 2B2 pertaining to BE-DBM it is possible to observe increased presence of fibrillar structures within the BE-DBM samples. The white arrows in Figure 2A2 and Figure 2B2 point to these structures. These structures represent the ECM deposition within the DBM.

### The BE-DBM promotes improved stem cell attachment and proliferation

**Figure 3:**
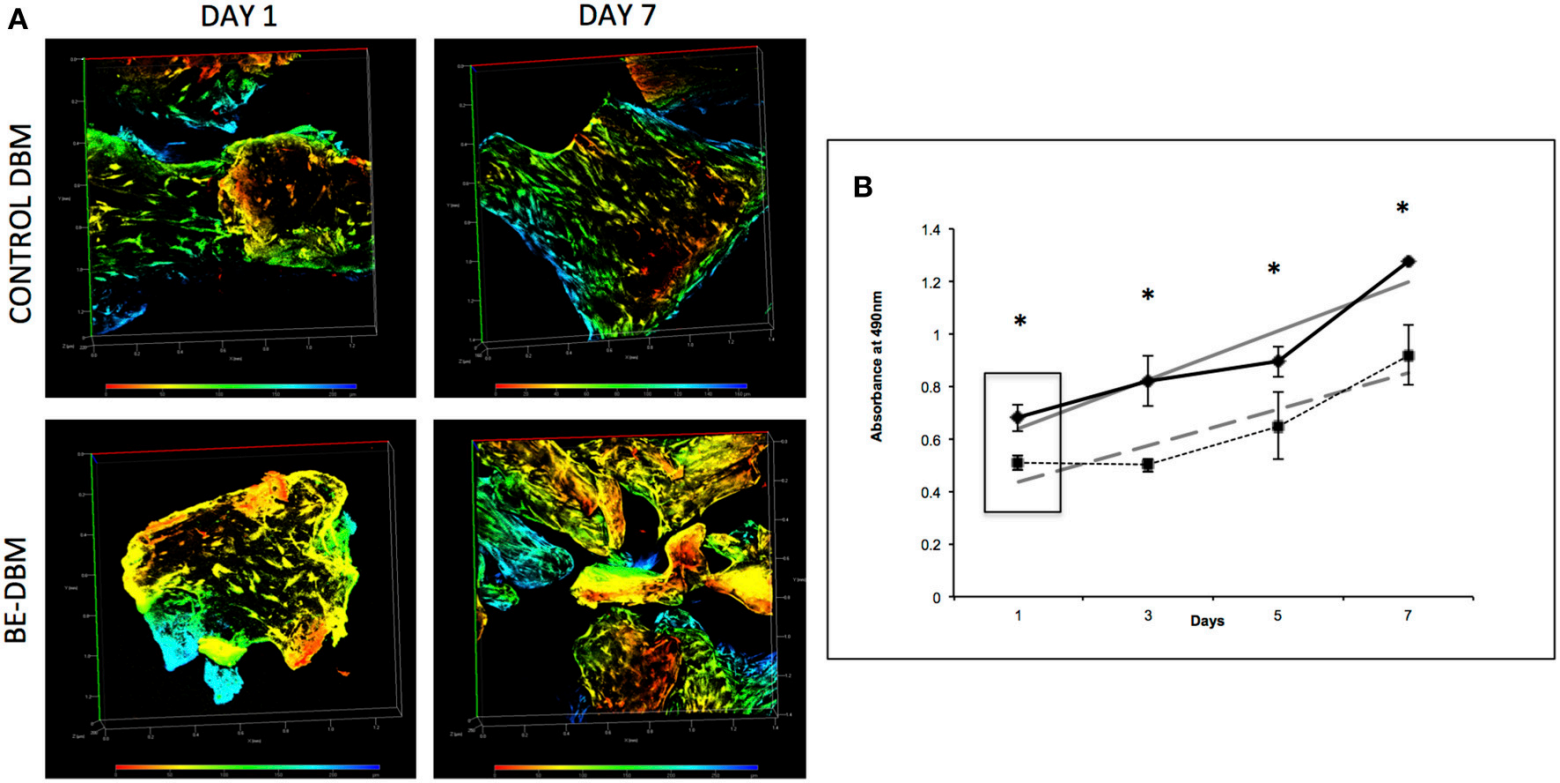
**Attachment and proliferation of HMSCs in DBM and BE-DBM**. **(A)** Represents 3D renderings of z-stack confocal images of HMSCs within DBM and BE-DBM after 1 day and 1 week. These images provide qualitative proof that the BE-DBM promotes enhanced stem cell attachment and proliferation. The color-coding in the renderings represents depth as indicated by the depth scale below. **(B)** Represents proliferation of HMSCs in the DBM (dotted lines) and BE-DBM (solid lines) over 1 week. The data points represent mean of quadruplicates with standard deviation as error. The slope of the linear lines (dotted lines for DBM and solid lines for BE-DBM) provides the rate of proliferation. HMSCs in the BE-DBM showed a 34.52% increase in proliferation rate. The boxed region in the graph represents data obtained at day 1. A 34.07% (*P* < 0.01 by student's *t*-test) increase in initial cell attachment was seen in BE-DBM with respect to DBM. The data at all time points was statistically significant with *P* < 0.05. ^*^above each point represents statistical significance.

An MTT assay was used to quantitate proliferation of HMSCs on DBM and BE-DBM. Results presented in Figure 3B show that the BE-DBM promotes improved stem cell attachment (boxed area representing the 1 day time point). A 34.07% (*P* < 0.01 by student's *t*-test) increase in cellular attachment was observed in the BE-DBM compared to DBM. Rate of proliferation was obtained by calculating the slope of the lines representing proliferation of the cells over a period of 1 week. The results showed that the BE-DBM enhanced the rate of proliferation of HMSCs by 34.52%.

Figure 3A shows representative images of HMSCs in DBM and BE-DBM after 1 day and 7 days. The images show qualitatively the increased presence of cells in the BE-DBM at these time points.

### The BE-DBM promotes improved osteogenic differentiation of HMSCs *in vitro*

**Figure 4:**
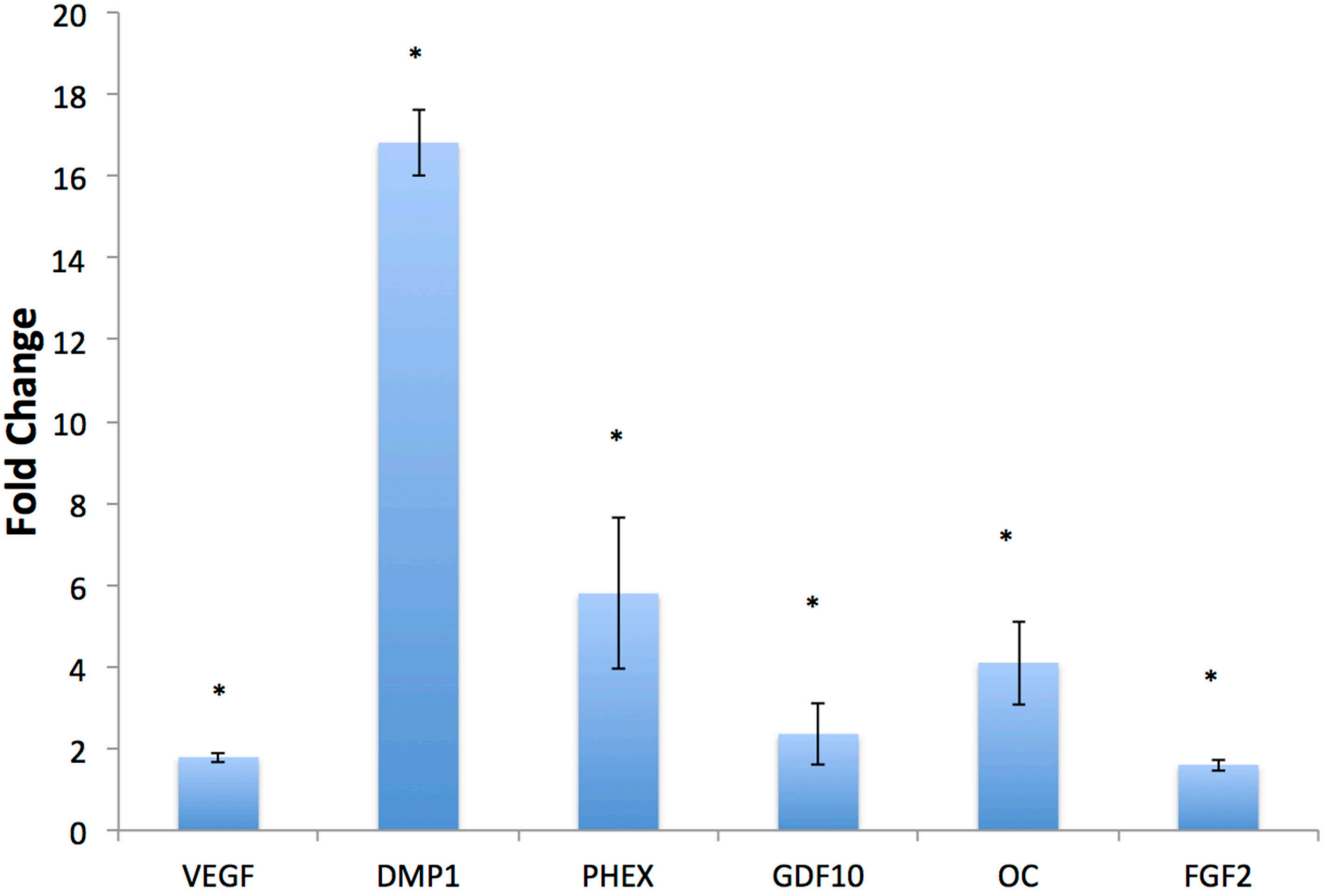
***In vitro* regulation of osteogenic genes**. The graph represents fold change in gene regulation of key pro-osteogenic genes and growth factors in BE-DBM with respect to control DBM. Data are represented as mean fold change obtained from quadruplicate experiments with standard deviation as error. Student's *t*-test used to obtain statistical significance with respect to control. ^*^Represents significance of *P* < 0.05.

HMSCs were cultured under normal growth conditions in both DBM and BE-DBM for 2 weeks. qPCR results presented in Figure 4 show a statistically significant change in the expression of several key pro-osteogenic proteins such as DMP1, phosphate regulating endopeptidase homolog X (PHEX) and osteocalcin. We also observed significant increase in the expression of osteogenic growth factors such as growth and differentiation factor 10 (GDF10) and FGF2 and pro angiogenic factor VEGF.

### The BE-DBM promotes improved matrix mineralization *in vitro*

**Figure 5:**
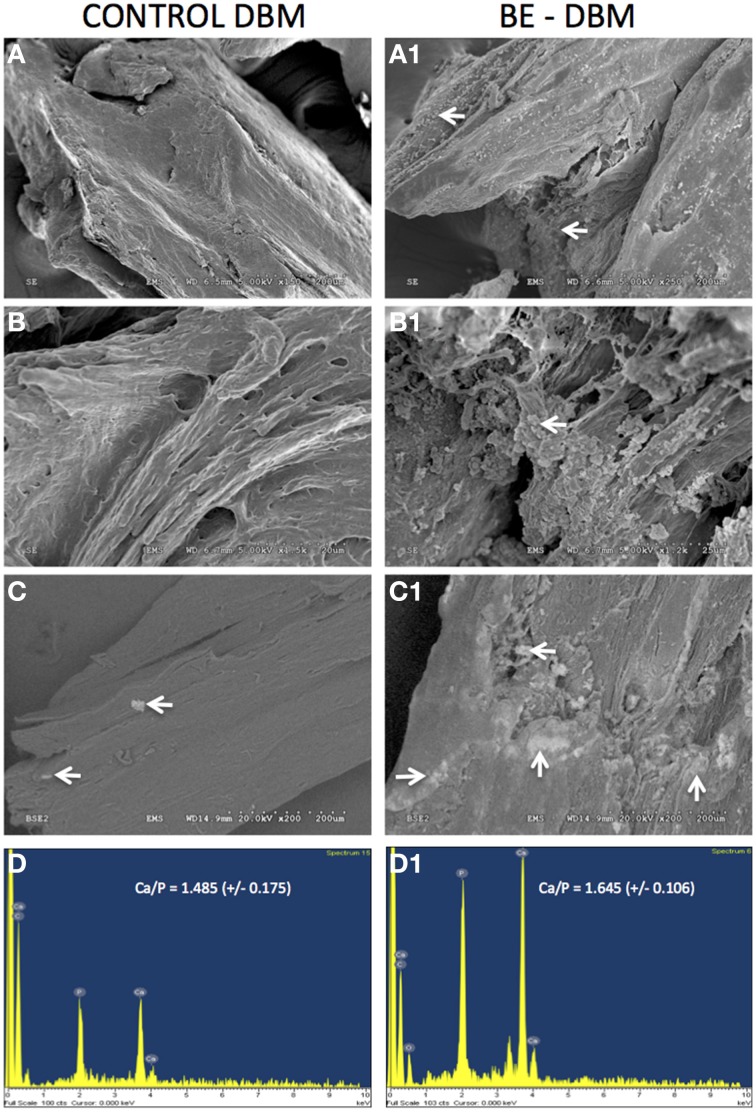
***In vitro* calcium phosphate nucleation**. Panels **(A–C)** are representative electron micrographs of DBM samples subjected to *in vitro* calcium phosphate nucleation. Panels **(A1–C1)** are electron micrographs of BE-DBM samples subjected to *in vitro* nucleation. Note the increased deposition on the BE-DBM samples. Panels **(C,C1)** were acquired using samples that were not coated with pt/pd. White arrows in **(C,C1)** indicate areas of calcium phosphate deposition. Panels **(D,D1)** are representative EDX spectrums of DBM and BE-DBM samples respectively showing the presence of calcium and phosphorus.

The ability of DBM and BE-DBM to promote nucleation of calcium phosphate crystals was evaluated using an *in vitro* nucleation experiment. Under these conditions, the DBM did not nucleate a large amount of calcium phosphate crystals. Results presented in Figures 5A,B show similar microstructure to DBM that was not subjected to *in vitro* nucleation experiment (Figures 2A,B). On the other hand, results presented in Figures 5A1,B1 show the presence several structures resembling nucleated calcium phosphate crystals. These images were obtained from pt/pd-coated samples of mineralized DBM and BE-DBM. In addition, imaging of mineralized DBM and BE-DBM was performed on uncoated samples in variable pressure mode. Results presented in Figure 5C show the presence of a few calcium phosphate crystals in the DBM samples. The bright areas in the image (white arrows) are calcium phosphate deposits. The presence of calcium and phosphorus was verified by EDX elemental analysis (Figure 5D). The ratio of calcium to phosphorus in these deposits was 1.485 (*S.D* = 0.175, *n* = 8).

On the other hand, results presented in Figure 5C1 show a very robust deposition of calcium phosphate throughout the BE-DBM sample (white arrows). Presence of calcium and phosphorus was verified using EDX analysis (Figure 5D1) and the ratio of calcium to phosphorus was 1.645 (*S.D* = 0.106, *n* = 8). The difference in the Calcium to phosphorus ratio between the two samples was statistically significant with a *P*-value of 0.033 using student's *t*-test.

### The BE-DBM promotes improved osteogenic differentiation *in vivo*

**Figure 6:**
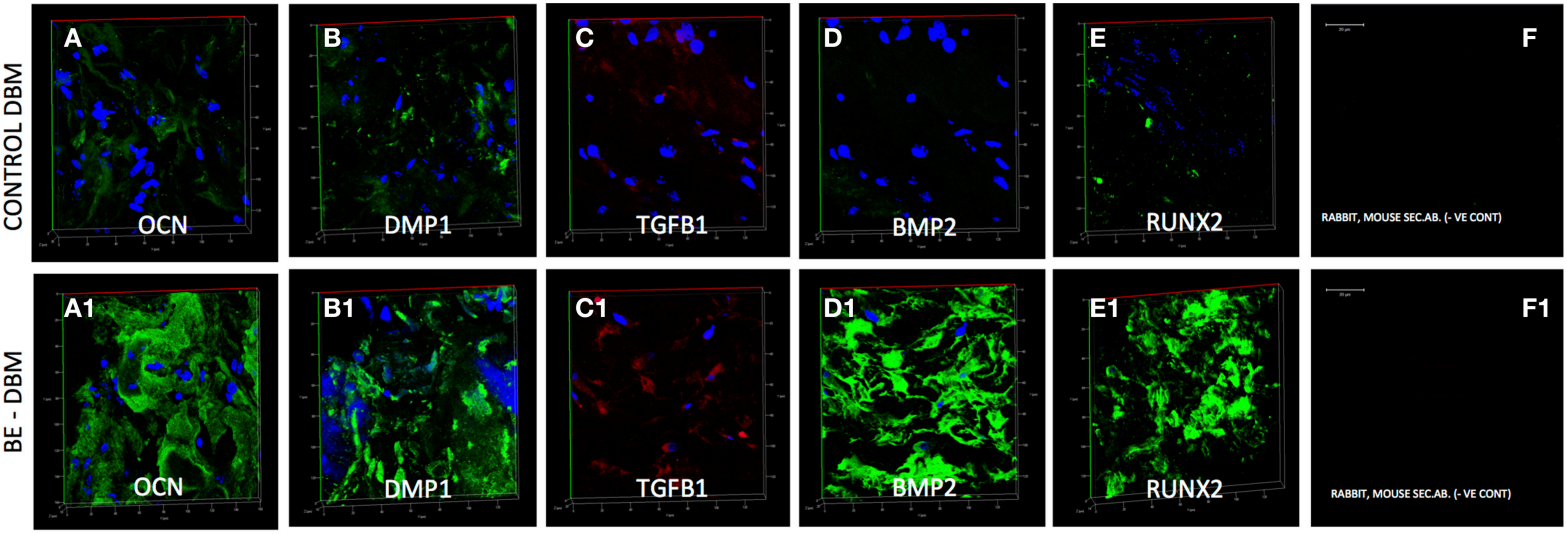
**Immunohistochemistry of DBM and BE-DBM *in vivo* explants**. All images except secondary antibody controls **(F,F1)** are 3D renderings of z-stack confocal images. Scale bar in **(F,F1)** represents 20 μm. The 3D images represent expression of osteocalcin (OCN, **A,A1**), DMP1 **(B,B1)**, TGFβ1 **(C,C1)**, BMP2 **(D,D1)**, and RUNX2 **(E,E1)** in DBM **(A–E)** and BE-DBM **(A1–E1)** samples after 2 weeks of subcutaneous implantation with HMSCs in immunocompromised mice. Note the marked increase in the expression levels of all the proteins in the BE-DBM.

Gene expression data is not valuable if protein expression does not follow *in vivo*. HMSCs were seeded on to DBM and BE-DBM, wrapped in clinical grade collagen sponges and implanted subcutaneously for 2 weeks in immunocompromised mice. Results presented in Figure 6 show that the HMSCs in BE-DBM expressed higher amounts of osteocalcin, DMP1, BMP2, TGFβ1, and RUNX2 when compared to those in commercial DBM.

### The BE-DBM promotes enhanced mineralization and vascularization *in vivo*

**Figure 7:**
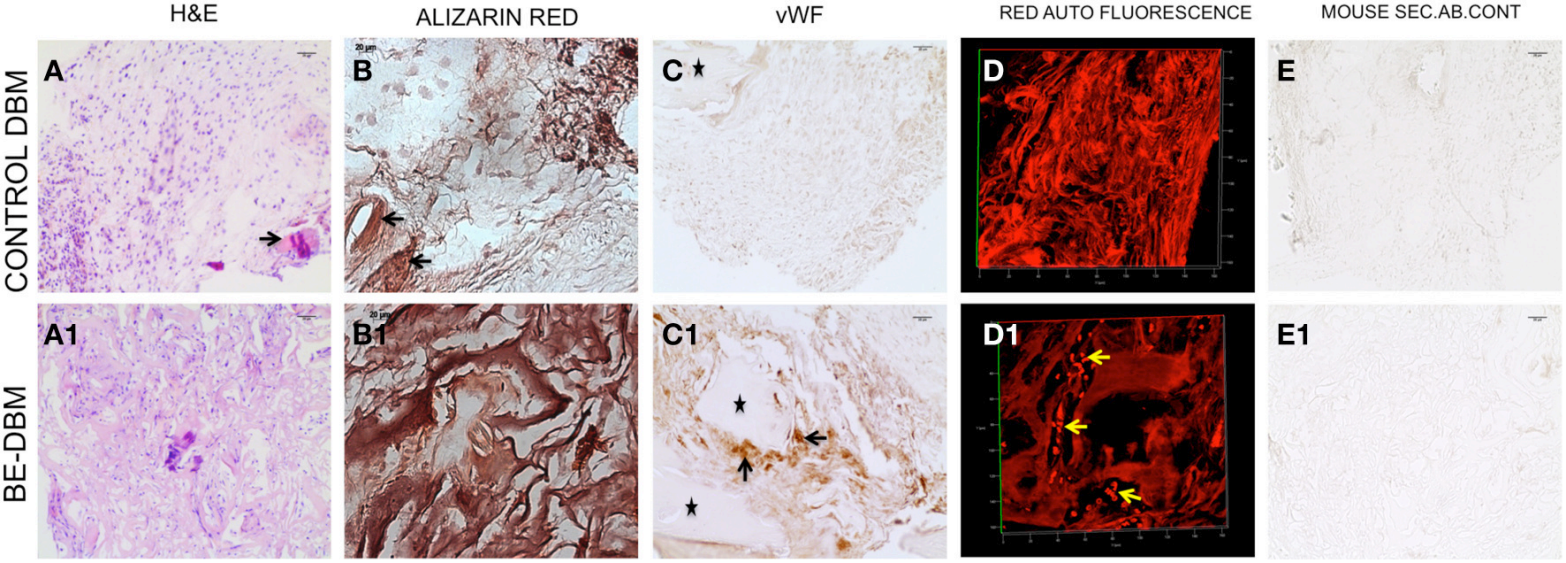
**Histology of DBM and BE-DBM *in vivo* explants**. **(A,A1)** Represent H&E stained sections of DBM and BE-DBM explants, respectively. The arrow in **(A)** points to a cortical bone chip. **(B,B1)** Represent alizarin red stained sections from DBM and BE-DBM explants. **(C,C1)** Represent DBM and BE-DBM sections immunohistochemically stained for von Willebrand factor. The arrows in **(C1)** point to positively stained endothelial cells in the BE-DBM section. ^*^Symbols in **(C,C1)** represent cortical bone chips. **(D,D1)** Represent 3D confocal images of red channel auto fluorescence from H&E stained DBM and BE-DBM sections. The yellow arrows in **(D1)** point to RBCs. **(E,E1)** Represent mouse secondary antibody controls for the DBM and BE-DBM sections. Scale bar in all other images represents 20 μm.

The key to successful bone regeneration is collagen deposition and matrix mineralization. The 2-week explant sections were stained with H&E to observe general tissue architecture. Results presented in Figures 7A,A1 show more robust eosin staining in the BE-DBM sections compared to DBM sections indicating a higher presence of collagen. Alizarin red staining was performed to look for calcium deposition. Results presented in Figures 7B,B1 show that the BE-DBM containing sections showed more robust staining with alizarin red indicating a higher calcium deposition in the matrix.

The DBM that was used for generating the BE-DBM and control DBM samples for *in vivo* experiments contained cortical bone chips. The cortical bone chips served as an internal control for collagen presence and calcium presence in natural bone. The black arrows in Figures 7A,A1,B,B1 point to cortical bone chips.

Angiogenesis is vital for bone regeneration. Our results indicate that the BE-DBM contains more VEGF compared to commercially available DBM (Figures 1E,E1). We analyzed if this translated to improved vascularization *in vivo*. The BE-DBM sections showed a higher amount of von Willebrand factor positive endothelial cells than the DBM sections (Figures 7C,C1) indicating that the BE-DBM promoted migration of host endothelial cells better than DBM. We quantified the von Willebrand factor staining. Six images spanning three samples for each group were used for this purpose. In the BE-DBM samples, on an average 10.53% of the area in the images stained positive for von Willebrand factor as opposed to 2.15% in the DBM samples. Student's *t*-test on the data showed a *P*-value of 0.0196 indicating that the data was statistically significant.

Additionally, the DBM samples did not show any endothelial cells within the samples. In certain areas, we were able to observe endothelial cells in the periphery of the sample. However, these may include some mouse connective tissue as well. We included these areas as well in our calculation. The ^*^ in Figures 7C,C1 indicate cortical bone chips. When the H&E stained sections were imaged using a confocal microscope, RBCs were observed only in the BE-DBM sections indicating active blood flow (Figures 7D,D1). This data corroborates well with the von Willebrand factor staining of the samples that indicated that there were no endothelial cells within the DBM samples. The presence of RBCs were not scattered, but concentrated in the shape of vessels (yellow arrows in Figure 7D) indicating that they were not from random bleeding. Finally, no secondary antibody non-specific staining was observed in our experiments (Figures 7E,E1).

## Discussion

The ECM is a dynamic environment that dictates cellular behavior and tissue functionality. We have published several articles on the use of decellularized ECM as a biomimetic biomaterial for tissue regeneration (Ravindran et al., 2012, 2014a,b). Decellularized biomimetic matrices have gained prominence recently and have been evaluated for several tissue engineering applications such as heart (Moroni and Mirabella, 2014), lung (Petersen et al., 2012; Calle et al., 2015), and cartilage (Schwarz et al., 2012). The advantage of these matrices over other biomaterials is that they can provide a tissue specific environment. Several studies have shown the effectiveness and safety of this process (Crapo et al., 2011). The limitation however, is the maintenance of tissue integrity (Moroni and Mirabella, 2014). In the case of DBM this is not an issue as the starting material itself is demineralized and decellularized bone particles.

In this study, we have characterized a biomimetically-enhanced version of clinical DBM that has the potential to significantly improve the quality of bone regenerative procedures. Our results show that the BE-DBM contains significantly more pro-osteogenic factors. One of the significant observations was the increased presence of fibronectin. Fibronectin binds several growth factors (Goerges and Nugent, 2004; Zhu and Clark, 2014). Therefore, an increase in fibronectin can result in increased sequestration of growth factors *in vivo*. Apart from fibronectin, growth factors such as VEGF, BMP2, and TGFβ1 were also observed in increased amounts in the BE-DBM compared to DBM. The BE-DBM also contained significantly higher amounts of phosphorylated proteins. Phosphorylated proteins serve as the source for inorganic phosphorus during hydroxyapatite nucleation. Therefore, the increased presence of these proteins was an encouraging sign that the BE-DBM may promote faster and better nucleation. Although the qualitative and quantitative data provided in this study is not an exhaustive list of proteins present in the matrix, they provide a good overview to summarize that the BE-DBM that we have generated contains significantly more pro-osteogenic proteins.

During bone regeneration, recruitment of MSCs to the site is critical for new bone formation. Therefore, a bone regenerative material should possess the ability to promote stem cell attachment and proliferation. The BE-DBM promoted improved stem cell attachment and proliferation compared to DBM indicating its enhanced potential to recruit stem cells. Additionally, the BE-DBM also promotes better osteogenic differentiation of MSCs compared to DBM.

Our results indicated statistically significant regulation of key pro-osteogenic genes. Our results indicated a significant increase in the expression levels of PHEX, DMP1 and OC. PHEX and DMP1 are extremely important for mineralized matrix formation and regulate FGF23 expression (Martin et al., 2011). PHEX is an endopeptidase that acts upon the mineralization inhibitor osteopontin to positively regulate mineralized matrix formation (Zhu and Clark, 2014). DMP1 is a multi-functional pro-osteogenic protein that possesses several intra and extracellular roles ranging from control of MSC differentiation to serving as a collagen binding protein that actively nucleates hydroxyapatite (He et al., 2003; He and George, 2004; Ravindran et al., 2008; Eapen et al., 2010). OC is one of the most abundant non-collagenous proteins in bone. OC acts as an endocrine hormone and plays a pivotal role in bone formation and increase in bone mineral density (Lee et al., 2007). Apart from these key regulators of bone formation, the BE-DBM also stimulated an increase in the expression levels of the pro-angiogenic growth factor VEGF and pro-osteogenic growth factors GDF10 (Kaihara et al., 2003; Marsell and Einhorn, 2009) and FGF2 (Hong et al., 2010; Kigami et al., 2013). These results indicated the enhanced potential of the BE-DBM to induce MSC differentiation.

When subjected to *in vitro* nucleation experiments to explore the calcium phosphate nucleating ability of the BE-DBM compared to DBM, the BE-DBM showed a more robust nucleating ability with calcium to phosphorus ratio similar to that of hydroxyapatite found in bone. Collectively, our *in vitro* experiments indicated that the BE-DBM is better than DBM in promoting stem cell attachment, proliferation, differentiation and hydroxyapatite nucleation.

When BE-DBM and DBM were compared *in vivo* in a subcutaneous implantation model with HMSCs, the BE-DBM promoted better collagen synthesis and formation of a better-calcified matrix as evidenced by our histological data. These observations are in line with our *in vitro* data that indicated the ability of the BE-DBM to promote these events better than DBM. Additionally, in line with the positive regulation of pro-angiogenic factors *in vitro*, the BE-DBM also promoted better migration of host endothelial cells and vascularization when compared to commercial DBM. Finally, fluorescence immunohistochemistry of the explant sections revealed that the BE-DBM was able to promote better osteogenic differentiation of MSCs by inducing higher levels of expression of pro-osteogenic non-collagenous proteins such as OC and DMP1, growth factors such as BMP2 and TGFβ1 (Yamamoto et al., 2000; Ozkan et al., 2007) and important pro-osteogenic transcription factors such as RUNX2 (Komori, 2008; Zhang et al., 2009). These results indicated that the BE-DBM accelerated osteogenic differentiation and matrix mineralization *in vivo*.

Overall, the data presented in this manuscript shows that the BE-DBM promotes better differentiation of HMSCs *in vitro* and *in vivo*. However, further investigation using large animal critical-size bone defect models is required to verify the improved osteoinductive properties of BE-DBM. Our efforts at present are focused on these models and if successful, this product can bring about standardization to the quality and performance of DBM that is critically lacking at present. DBM is widely used in craniofacial bone regeneration, spinal fusion and in non-union fracture healing in conjunction with autologous bone (Gruskin et al., 2012). Based on our results, we predict that the BE-DBM will enable faster regeneration of higher quality bone in these applications.

This is the first report that shows that it is possible to generate a biomimetic ECM incorporated DBM that has the potential replace the existing material. The amount of ECM incorporated within DBM to generate BE-DBM can be standardized by the use of cell lines and by controlling the cell number, density and culture conditions using a bioreactor. If these conditions are standardized, it is feasible to scale up the production of BE-DBM for commercial use.

### Conflict of interest statement

The authors declare that the research was conducted in the absence of any commercial or financial relationships that could be construed as a potential conflict of interest.

## References

[B1] Bae H. Zhao L. Zhu D. Kanim L. E. Wang J. C. Delamarter R. B. (2010). Variability across ten production lots of a single demineralized bone matrix product. J. Bone Joint Surg. Am. 92, 427–435. 10.2106/JBJS.H.01400 20124070

[B2] Bae H. W. Zhao L. Kanim L. E. Wong P. Delamarter R. B. Dawson E. G. (2006). Intervariability and intravariability of bone morphogenetic proteins in commercially available demineralized bone matrix products. Spine (Phila Pa 1976) 31, 1299–1306. discussion 1307–1298. 10.1097/01.brs.0000218581.92992.b7 16721289

[B3] Calle E. A. Mendez J. J. Ghaedi M. Leiby K. L. Bove P. F. Herzog E. L.. (2015). Fate of distal lung epithelium cultured in a decellularized lung extracellular matrix. Tissue Eng. Part A 21, 1916–1928. 10.1089/ten.TEA.2014.0511 25789725 PMC4449714

[B4] Crapo P. M. Gilbert T. W. Badylak S. F. (2011). An overview of tissue and whole organ decellularization processes. Biomaterials 32, 3233–3243. 10.1016/j.biomaterials.2011.01.057 21296410 PMC3084613

[B5] Delloye C. Cornu O. Druez V. Barbier O. (2007). Bone allografts: what they can offer and what they cannot. J. Bone Joint Surg. Br. 89, 574–579. 10.1302/0301-620X.89B5.19039 17540738

[B6] Di Bella C. Dozza B. Frisoni T. Cevolani L. Donati D. (2010). Injection of demineralized bone matrix with bone marrow concentrate improves healing in unicameral bone cyst. Clin. Orthop. Relat. Res. 468, 3047–3055. 10.1007/s11999-010-1430-5 20568027 PMC2947677

[B7] Drosos G. I. Touzopoulos P. Ververidis A. Tilkeridis K. Kazakos K. (2015). Use of demineralized bone matrix in the extremities. World J. Orthop. 6, 269–277. 10.5312/wjo.v6.i2.269 25793167 PMC4363809

[B8] Eapen A. Sundivakkam P. Song Y. Ravindran S. Ramachandran A. Tiruppathi C.. (2010). Calcium-mediated stress kinase activation by DMP1 promotes osteoblast differentiation. J. Biol. Chem. 285, 36339–36351. 10.1074/jbc.M110.145607 20841352 PMC2978562

[B9] George A. Ravindran S. (2010). Protein templates in hard tissue engineering. Nano Today 5, 254–266. 10.1016/j.nantod.2010.05.005 20802848 PMC2928485

[B10] Goerges A. L. Nugent M. A. (2004). pH regulates vascular endothelial growth factor binding to fibronectin: a mechanism for control of extracellular matrix storage and release. J. Biol. Chem. 279, 2307–2315. 10.1074/jbc.M308482200 14570917

[B11] Gruskin E. Doll B. A. Futrell F. W. Schmitz J. P. Hollinger J. O. (2012). Demineralized bone matrix in bone repair: history and use. Adv. Drug Deliv. Rev. 64, 1063–1077. 10.1016/j.addr.2012.06.008 22728914 PMC7103314

[B12] Han B. Woodell-May J. Ponticiello M. Yang Z. Nimni M. (2009). The effect of thrombin activation of platelet-rich plasma on demineralized bone matrix osteoinductivity. J. Bone Joint Surg. Am. 91, 1459–1470. 10.2106/JBJS.H.00246 19487525

[B13] He G. Dahl T. Veis A. George A. (2003). Nucleation of apatite crystals *in vitro* by self-assembled dentin matrix protein 1. Nat. Mater. 2, 552–558. 10.1038/nmat945 12872163

[B14] He G. George A. (2004). Dentin matrix protein 1 immobilized on type I collagen fibrils facilitates apatite deposition *in vitro*. J. Biol. Chem. 279, 11649–11656. 10.1074/jbc.M309296200 14699165

[B15] Hong K. S. Kim E. C. Bang S. H. Chung C. H. Lee Y. I. Hyun J. K.. (2010). Bone regeneration by bioactive hybrid membrane containing FGF2 within rat calvarium. J. Biomed. Mater. Res. A 94, 1187–1194. 10.1002/jbm.a.32799 20694985

[B16] Horváthy D. B. Vácz G. Toró I. Szabó T. May Z. Duarte M.. (2015). Remineralization of demineralized bone matrix in critical size cranial defects in rats: a 6-month follow-up study. J. Biomed. Mater. Res. B Appl. Biomater. [Epub ahead of print]. 10.1002/jbm.b.33446 26138348

[B17] Kaihara S. Bessho K. Okubo Y. Sonobe J. Komatsu Y. Miura M.. (2003). Over expression of bone morphogenetic protein-3b (BMP-3b) using an adenoviral vector promote the osteoblastic differentiation in C2C12 cells and augment the bone formation induced by bone morphogenetic protein-2 (BMP-2) in rats. Life Sci. 72, 1683–1693. 10.1016/S0024-3205(02)02477-3 12559390

[B18] Kigami R. Sato S. Tsuchiya N. Yoshimakai T. Arai Y. Ito K. (2013). FGF-2 angiogenesis in bone regeneration within critical-sized bone defects in rat calvaria. Implant Dent. 22, 422–427. 10.1097/ID.0b013e31829d19f0 23835540

[B19] Komori T. (2008). Regulation of bone development and maintenance by Runx2. Front. Biosci. 13, 898–903. 10.2741/2730 17981598

[B20] Lee N. K. Sowa H. Hinoi E. Ferron M. Ahn J. D. Confavreux C.. (2007). Endocrine regulation of energy metabolism by the skeleton. Cell 130, 456–469. 10.1016/j.cell.2007.05.047 17693256 PMC2013746

[B21] Lohmann C. H. Andreacchio D. Köster G. Carnes D. L. Jr. Cochran D. L. Dean D. D.. (2001). Tissue response and osteoinduction of human bone grafts *in vivo*. Arch. Orthop. Trauma Surg. 121, 583–590. 10.1007/s004020100291 11768641

[B22] Marino J. T. Ziran B. H. (2010). Use of solid and cancellous autologous bone graft for fractures and nonunions. Orthop. Clin. North Am. 41, 15–26; table of contents. 10.1016/j.ocl.2009.08.003 19931049

[B23] Marsell R. Einhorn T. A. (2009). The role of endogenous bone morphogenetic proteins in normal skeletal repair. Injury 40(Suppl. 3), S4–S7. 10.1016/S0020-1383(09)70003-8 20082790

[B24] Martin A. Liu S. David V. Li H. Karydis A. Feng J. Q.. (2011). Bone proteins PHEX and DMP1 regulate fibroblastic growth factor Fgf23 expression in osteocytes through a common pathway involving FGF receptor (FGFR) signaling. FASEB J. 25, 2551–2562. 10.1096/fj.10-177816 21507898 PMC3136343

[B25] Moroni F. Mirabella T. (2014). Decellularized matrices for cardiovascular tissue engineering. Am. J. Stem Cells 3, 1–20. 24660110 PMC3960753

[B26] Ozkan K. Eralp L. Kocaoglu M. Ahishali B. Bilgic B. Mutlu Z.. (2007). The effect of transforming growth factor beta1 (TGF-beta1) on the regenerate bone in distraction osteogenesis. Growth Factors 25, 101–107. 10.1080/08977190701352594 17891595

[B27] Park I. H. Micic I. D. Jeon I. H. (2008). A study of 23 unicameral bone cysts of the calcaneus: open chip allogeneic bone graft versus percutaneous injection of bone powder with autogenous bone marrow. Foot Ankle Int. 29, 164–170. 10.3113/FAI.2008.0164 18315971

[B28] Petersen T. H. Calle E. A. Colehour M. B. Niklason L. E. (2012). Matrix composition and mechanics of decellularized lung scaffolds. Cells Tissues Organs 195, 222–231. 10.1159/000324896 21502745 PMC3696368

[B29] Pietrzak W. S. Woodell-May J. McDonald N. (2006). Assay of bone morphogenetic protein-2, -4, and -7 in human demineralized bone matrix. J. Craniofac. Surg. 17, 84–90. 10.1097/01.scs.0000179745.91165.73 16432413

[B30] Ravindran S. Gao Q. Kotecha M. Magin R. L. Karol S. Bedran-Russo A.. (2012). Biomimetic extracellular matrix-incorporated scaffold induces osteogenic gene expression in human marrow stromal cells. Tissue Eng. Part A 18, 295–309. 10.1089/ten.TEA.2011.0136 21867449 PMC3267968

[B31] Ravindran S. Huang C. C. George A. (2014a). Extracellular matrix of dental pulp stem cells: applications in pulp tissue engineering using somatic MSCs. Front. Physiol. 4:395. 10.3389/fphys.2013.00395 24432005 PMC3880843

[B32] Ravindran S. Kotecha M. Huang C. C. Ye A. Pothirajan P. Yin Z.. (2015). Biological and MRI characterization of biomimetic ECM scaffolds for cartilage tissue regeneration. Biomaterials 71, 58–70. 10.1016/j.biomaterials.2015.08.030 26318817 PMC4572912

[B33] Ravindran S. Narayanan K. Eapen A. S. Hao J. Ramachandran A. Blond S.. (2008). Endoplasmic reticulum chaperone protein GRP-78 mediates endocytosis of dentin matrix protein 1. J. Biol. Chem. 283, 29658–29670. 10.1074/jbc.M800786200 18757373 PMC2573078

[B34] Ravindran S. Zhang Y. Huang C. C. George A. (2014b). Odontogenic induction of dental stem cells by extracellular matrix-inspired three-dimensional scaffold. Tissue Eng. Part A 20, 92–102. 10.1089/ten.TEA.2013.0192 23859633 PMC3875192

[B35] Schwartz Z. Somers A. Mellonig J. T. Carnes D. L. Jr. Dean D. D. Cochran D. L.. (1998). Ability of commercial demineralized freeze-dried bone allograft to induce new bone formation is dependent on donor age but not gender. J. Periodontol. 69, 470–478. 10.1902/jop.1998.69.4.470 9609378

[B36] Schwarz S. Koerber L. Elsaesser A. F. Goldberg-Bockhorn E. Seitz A. M. Dürselen L.. (2012). Decellularized cartilage matrix as a novel biomatrix for cartilage tissue-engineering applications. Tissue Eng. Part A 18, 2195–2209. 10.1089/ten.TEA.2011.0705 22690787

[B37] Sekiya I. Larson B. L. Smith J. R. Pochampally R. Cui J. G. Prockop D. J. (2002). Expansion of human adult stem cells from bone marrow stroma: conditions that maximize the yields of early progenitors and evaluate their quality. Stem Cells 20, 530–541. 10.1634/stemcells.20-6-530 12456961

[B38] Soicher M. A. Christiansen B. A. Stover S. M. Leach J. K. Fyhrie D. P. (2013). Remineralization of demineralized bone matrix (DBM) via alternating solution immersion (ASI). J. Mech. Behav. Biomed. Mater. 26, 109–118. 10.1016/j.jmbbm.2013.05.007 23759125 PMC3713112

[B39] Thibault R. A. Scott Baggett L. Mikos A. G. Kasper F. K. (2010). Osteogenic differentiation of mesenchymal stem cells on pregenerated extracellular matrix scaffolds in the absence of osteogenic cell culture supplements. Tissue Eng. Part A 16, 431–440. 10.1089/ten.TEA.2009.0583 19863274 PMC2813072

[B40] Traianedes K. Russell J. L. Edwards J. T. Stubbs H. A. Shanahan I. R. Knaack D. (2004). Donor age and gender effects on osteoinductivity of demineralized bone matrix. J. Biomed. Mater. Res. B Appl. Biomater. 70, 21–29. 10.1002/jbm.b.30015 15199579

[B41] Yamamoto M. Tabata Y. Hong L. Miyamoto S. Hashimoto N. Ikada Y. (2000). Bone regeneration by transforming growth factor beta1 released from a biodegradable hydrogel. J. Control. Release 64, 133–142. 10.1016/S0168-3659(99)00129-7 10640652

[B42] Zara J. N. Siu R. K. Zhang X. Shen J. Ngo R. Lee M.. (2011). High doses of bone morphogenetic protein 2 induce structurally abnormal bone and inflammation *in vivo*. Tissue Eng. Part A 17, 1389–1399. 10.1089/ten.TEA.2010.0555 21247344 PMC3079169

[B43] Zhang M. Powers R. M. Jr. Wolfinbarger L. Jr. (1997). Effect(s) of the demineralization process on the osteoinductivity of demineralized bone matrix. J. Periodontol. 68, 1085–1092. 10.1902/jop.1997.68.11.1085 9407401

[B44] Zhang S. Xiao Z. Luo J. He N. Mahlios J. Quarles L. D. (2009). Dose-dependent effects of Runx2 on bone development. J. Bone Miner. Res. 24, 1889–1904. 10.1359/jbmr.090502 19419310 PMC2765932

[B45] Zhu J. Clark R. A. (2014). Fibronectin at select sites binds multiple growth factors and enhances their activity: expansion of the collaborative ECM-GF paradigm. J. Invest. Dermatol. 134, 895–901. 10.1038/jid.2013.484 24335899 PMC3961531

[B46] Zuk P. A. (2008). Tissue engineering craniofacial defects with adult stem cells? Are we ready yet? Pediatr. Res. 63, 478–486. 10.1203/PDR.0b013e31816bdf36 18427291

